# EigenTHREADER: analogous protein fold recognition by efficient contact map threading

**DOI:** 10.1093/bioinformatics/btx217

**Published:** 2017-04-13

**Authors:** Daniel W A Buchan, David T Jones

**Affiliations:** Department of Computer Science, University College London, Gower Street, London, UK

## Abstract

**Motivation:**

Protein fold recognition when appropriate, evolutionarily-related, structural templates can be identified is often trivial and may even be viewed as a solved problem. However in cases where no homologous structural templates can be detected, fold recognition is a notoriously difficult problem (Moult *et al.*, 2014). Here we present EigenTHREADER, a novel fold recognition method capable of identifying folds where no homologous structures can be identified. EigenTHREADER takes a query amino acid sequence, generates a map of intra-residue contacts, and then searches a library of contact maps of known structures. To allow the contact maps to be compared, we use eigenvector decomposition to resolve the principal eigenvectors these can then be aligned using standard dynamic programming algorithms. The approach is similar to the Al-Eigen approach of Di Lena *et al.* (2010), but with improvements made both to speed and accuracy. With this search strategy, EigenTHREADER does not depend directly on sequence homology between the target protein and entries in the fold library to generate models. This in turn enables EigenTHREADER to correctly identify analogous folds where little or no sequence homology information is.

**Results:**

EigenTHREADER outperforms well-established fold recognition methods such as pGenTHREADER and HHSearch in terms of True Positive Rate in the difficult task of analogous fold recognition. This should allow template-based modelling to be extended to many new protein families that were previously intractable to homology based fold recognition methods.

**Availability and implementation:**

All code used to generate these results and the computational protocol can be downloaded from https://github.com/DanBuchan/eigen_scripts. EigenTHREADER, the benchmark code and the data this paper is based on can be downloaded from: http://bioinfadmin.cs.ucl.ac.uk/downloads/eigenTHREADER/.

## 1 Introduction

Accurate prediction of protein structure from protein sequence remains a significant open problem in structural biology and bioinformatics, and this topic has received a great deal of attention in the preceding 50 years. While some sub-problems such as homology modelling have shown marked successes, progress for other aspects has remained relatively modest. A single, integrated mathematical model of protein folding remains elusive (Mitchell and Gronenborn, 2015).

Today, protein structure prediction typically proceeds by one of two broad strategies. Template-free or *ab initio* folding attempts to fold proteins using only the physiochemical information implicit in the protein sequence itself. To date, such methods have achieved rather limited success (Moult *et al.*, 2014), though recent developments in protein contact prediction are very promising. The alternative strategy, template based (or homology) modelling, is widely used by biologists as it has proven to be a robust predictive strategy, enjoying increasing success as both the sequence and structure databases expand.

Template based modelling proceeds by first attempting to identify suitable structural templates for the given query protein sequence. This initial step is commonly referred to as fold recognition. If one or more templates can be identified, the 3D structure or structures can then be used as the basis for homology modelling which will result in a predicted structure (Söding and Remmert, 2011).

As such, template-based modelling depends critically on successful fold recognition and to this end many sophisticated fold recognition strategies have been developed. Popular methods make use of computational methods as diverse as: dynamic programming, Support Vector Machines, neural networks, Hidden Markov Models, profile-profile comparison and so forth (Rost *et al.*, 1997; Olmea *et al.*, 1999; Zhou and Zhou, 2005; Wu and Zhang, 2008; Lobley *et al.*, 2009; Peng and Xu, 2011; Ma *et al.*, 2013; Gniewek *et al.*, 2014).

Fold recognition strategies often involve matching a query sequence against a representative library of known, possible template folds. The fold library is expressed in terms of physiochemical features such as secondary structure and solvent accessibility, which are easy to calculate for each template fold and will also, ideally, be easy to predict from the query sequence and its homologous sequences. Typically, each feature will be expressed as a vector over the length of each fold library member and the query sequence. This representation makes it easy to match the feature vectors of the query sequence to those in the fold library in a computationally efficient manner. With an appropriate scoring function, the quality of each match can in turn be assessed. Query-sequence to specific-fold matches which fulfil some given selection criteria will then be used as structural templates for further structural modelling procedures. Selection criteria vary in sophistication from simple heuristics (‘top *n* matches’) to probabilistic scoring using Neural Networks or Support Vector Machines.

Despite many successes, the early promise of classical threading methods, to detect protein folds in the absence of sequence similarity, has not stood the test of time (i.e. Jones *et al.*, 1992), or rather has not kept pace with the growth in both sequence and structure data banks. The basic idea of classical threading approaches was to use amino acid pair and solvation potentials to both pick out the best templates and find the optimal alignment (or threading). As fold space became more crowded, it became clear that these potentials alone were not sufficient. Present day methods combine features such as statistical potentials with sensitive sequence profile methods, which have become very powerful due to the exponential growth of sequence data banks, and it is these hybrid approaches that have come to dominate the field. Unfortunately, in cases where there is fold similarity but no evidence of common ancestry (so called analogous folds), sequence-directed fold recognition methods fail to provide adequate results. Here we present a new approach to protein fold recognition, called EigenTHREADER, which revisits the idea of detecting analogous folds by protein threading by exploiting new developments in residue-residue contact prediction rather than statistical potentials.

It has long been understood that protein structure can be accurately reconstructed when complete (or sufficient high quality) contact or distance constraint information is available. Indeed, this insight is the basis of solving protein structures by NMR data (Creighton, 1992).

With even sparse distance constraints, fold recognition is possible, even when high resolution structure reconstruction may not be possible. This is especially the case when the contact data available principally describes contacts between distal residues in the protein chain. It follows then, that if we can access or predict sufficient distance restraints from amino acid sequence, the fold of any given protein may be elucidated.

For many years only modest progress had been made in the problem of protein structure prediction via residue-residue contact prediction. However, recent substantial advances in accurate contact prediction, via co-evolutionary sequence analysis, have now rendered contact prediction a viable path to both *de novo* protein structure prediction and fold recognition (Marks *et al.*, 2011; Jones *et al.*, 2012; Kaján *et al.*, 2014; Kosciolek and Jones, 2014; Seemayer *et al.*, 2014; Jones *et al.*, 2015). We also note such advances have also allowed the development of highly accurate profile search methods such as MRFAlign (Ma *et al.*, 2014) which integrate both query sequence profile and contact data.

In this paper we present EigenTHREADER, a novel method for fold recognition which combines standard threading techniques with accurate contact prediction constraints. Predicted contact maps for query sequences are searched against a pre-generated library of contact maps representing possible template structures. EigenTHREADER has been specifically developed to tackle fold recognition problems in instances where powerful homology-driven detection methods such as HHSearch/HHPred (Söding, 2005) fail to produce results.

## 2 Materials and methods

### 2.1 Background

#### 2.1.1 Representation of a protein as a contact map

A protein’s 3D structure can be described in terms of its inter-residue contacts. A contact indicates that a pair of residues (ideally distant in primary sequence) lie close to one another in 3D space in the native folded tertiary structure. Where ‘close’ is defined by some given distance threshold. Typically, this distance threshold is set such that any two residues within the threshold distance may be assumed to take part in some form of physiochemical interaction. The underlying assumption being that such interactions may be critical to stabilizing the 3D structure of the protein. Interaction threshold distances are typically considered between 6 and 16 Å between the *C_α_* or *C_β_* carbons of the residue pairs. Given a threshold distance, a contact map (or matrix) can be constructed, which is a 2D representation of the inter-residue contacts within the tertiary structure of a protein chain. Contact maps are square, binary, symmetric matrices valued such that contacting residues are designated 1 and positions in the matrix which do not represent contacts take the value 0. When analysing contact maps adjacent residues are typically excluded or not considered in subsequent analysis as such contacts are trivially true under all contact distance thresholds due to simple amino-acid main-chain connectivity.

#### 2.1.2 The maximum contact map overlap (CMO) problem

The CMO problem asks, given two proteins (*P_1_* and *P_2_*) and their respective contact maps ($M^{P_{1}}\text{and}M^{P_{2}}$), what is the alignment of the 2D contact maps which maximizes the overlaps between the maps (i.e. best superimposes the two maps)? The problem is constrained such that positions in the first or second protein can be aligned with at most one position in the other protein. Any non-aligned positions are assumed to align to gaps. A second constraint requires that the ordering of residues in both sequences must be preserved.

Following on from the work of Di Lena *et al.* (2010) we reproduce here their formalization of the maximum CMO between two contact maps: The maximum CMO of $M^{P_{1}}\text{and}M^{P_{2}}$ can be calculated as: the alignment of two contact maps, f, which maximizes the quantity:

(1) OMP1,MP2=∑fi≠∅≠fjj>i+1,fj>fi+1MijP1·Mf(i)f(j)P2

Note that contacts between consecutive amino acids are not counted and that there is no penalty to the score for aligning a contact position in one matrix with a non-contact position in the other matrix. So, the maximum CMO is the alignment of the two matrices where the sum of the number of superimposed 1-valued elements is greatest.

### 2.2 EigenTHREADER

EigenTHREADER is a threading method which efficiently searches a library of protein folds (expressed as contact maps) with the contact map of a query protein. Contacts in the query contact map may be derived by experimental means (e.g. inferred from NMR or x-ray crystallographic data) or, of more relevance to this study, may be generated by predictive methods. In this study we make use of predicted contacts generated by MetaPSICOV (see Section 2.3). This method was found to be the most accurate contact prediction method in the most recent CASP experiment (Kinch *et al.*, 2016), and is thus an obvious starting point for contact threading. The maximum contact map overlap (CMO) between the predicted contact map for the query protein and every contact map in the fold library is calculated and scored. The highest scoring pairs can then be regarded as valid fold predictions for the query sequence as for those pairs the number of satisfied contacts is maximized.

Calculating the maximum CMO is known to be an NP hard problem (Goldman, 1999). EigenTHREADER calculates near maximal CMOs using the heuristic method, Al-Eigen, developed by Di Lena *et al.* (2010). We introduce some algorithmic improvements so that a large library of folds can be searched in reasonable time. The Al-Eigen method uses eigendecomposition of symmetric matrices (Strang, 2016) and the Needleman-Wunsch alignment algorithm (Needleman and Wunsch, 1970) to achieve high quality contact map alignments in polynomial time.

#### 2.2.1 Al-eigen

Here we briefly outline the Al-Eigen method, for a detailed treatment of the method we refer readers to the paper of Di Lena *et al.*

Eigendecomposition allows us to decompose any real-valued n × n symmetric matrix, *M*, into a series of eigenvectors and their associated eigenvalues. The matrix, *M*, can then be reconstituted by summing the outer product of each eigenvector-eigenvalue pair. It follows from this that the matrix *M* may be approximated, $\overset{-}{M}$, by considering only the few (*t*th) eigenvectors with the largest associated eigenvalues. Such that:

(2) $$\bar{M}=\sum_{i=1}^{t}\lambda_{i}(v_{i}⊗v_{i})$$

where $\bar{M}$ is the approximation of matrix *M* to order *t*, $v_{i}$ is the *i*th eigenvector and $\lambda_{i}$ is its associated eigenvalue. ⊗ denotes the outer product of the eigenvector to itself.

Two proteins can then be compared by considering the global alignment of the contact map eigenvectors rather than attempting to align the contact maps directly. This can be trivially computed in polynomial time with the Needleman-Wunsch algorithm given a scoring matrix with a specified gap penalty. Di Lena *et al.* state that their scoring function:

(3) $$S_{ij}=\sum_{k=1}^{t}{\left({u'}_{k}\right)}_{i}{\left({v'}_{k}\right)}_{j}$$

Assigns high scores where the entries in each eigenvector, ***u****′* and ***v****′*, have the same sign rather than the similar values.

#### 2.2.2 Efficient contact map search

The original Al-Eigen algorithm paper clearly showed that the quality of the alignments was seen to increase as the number of included eigenvectors was increased. However, due to the requirement in their algorithm to evaluate all possible eigenvector signs (as $v_{i}⊗v_{i}={-v}_{i}⊗{-v}_{i}$), the time required for each comparison scaled at 2^*n*^, where *n* is the maximum number of eigenvectors considered. This meant that in any practical search time, only a relatively small number of eigenvectors could be considered, limiting the accuracy of alignments.

Rather than exhaustively enumerating all possible eigenvector signs, EigenTHREADER opts instead for an iterative search procedure where we attempt to invert the signs of each eigenvector in turn, starting with the eigenvector associated with the largest eigenvalue. The CMO score is then assessed after each inversion, and any sign inversion which decreases the CMO score is rejected. Once a sign inversion is accepted, this process is repeated, starting again with the largest eigenvalue/eigenvector, until no further improvement in CMO score is observed. This modified algorithm is expected to scale by *n*^2^ rather than the 2^*n*^ of the original Al-Eigen. Although this iterative procedure cannot be guaranteed to produce optimal scores we have observed that it always achieves better alignments than Al-Eigen for any comparable runtime (data not shown).

As a further constraint to the alignment, a secondary structure matching score can also be optionally added to the CMO score matrix, up-weighting regions of the alignment path matrix where the predicted secondary structure of the target matches the observed secondary structure in the template.

#### 2.2.3 Final scoring

After the optimal contact map alignment is found, a final match score is produced by calculating the Pearson correlation coefficient between the MetaPSICOV contact probabilities and the contact distances in the template protein. One advantage of this score over other metrics is that it can be transformed easily into a t-statistic and so significance can be tested using a standard t-test. This provides a simple statistical significance test for contact map matches. Rather than using the t-statistic alone, as a final refinement of the scoring function, a logistic regression function is fitted to three variables: the t-statistic value, the fraction of the target that is aligned, and the fraction of the template that is aligned. The data used for this regression are pairwise matches (i.e. matching SCOP folds) in the MetaPSICOV (Jones *et al.*, 2015) training set, which does not overlap with the 150 test proteins. After the regression, this simple model gives good estimates of the probability of a fold-level match being correct for each matched template.

### 2.3 MetaPSICOV

For the EigenTHREADER performance benchmarking, query protein contacts were predicted using MetaPSICOV (Jones *et al.*, 2015). MetaPSICOV is a 2 stage neural network protein contact predictor which integrates contact predictions from multiple co-evolutionary protein contact predictors; PSICOV (Jones *et al.*, 2012), mfDAC/FreeContact (Kaján *et al.*, 2014) and CCMpred (Seemayer *et al.*, 2014).

In the first stage 672 features are generated for the prediction target protein. These cover a variety of physio-chemical properties such as solvation potential, helix-strand propensities, amino-acid propensities and sequence separation. Critically 6 input features are derived from the three contact prediction methods PSICOV, FreeContact and CCMpred. This stage outputs a predicted contact map for the query sequence.

The second stage neural network correlates the outputs for the first stage network analysing the predicted contact map from stage one. Taking an 11 × 11 window of the contact map this stage detects patterns to eliminate outlying predictions and infill gaps in the contact map. Inter-residue interactions such as main-chain hydrogen bonding are also identified at this stage. The second stage utilizes a superset of the first stage features with a total feature set of 731 features. Interested readers should refer to the MetaPSICOV paper and its Supplementary material (Jones *et al.*, 2015).

### 2.4 Benchmark data

150 single chain, single domain proteins with their associated predicted contacts were taken from the MetaPSICOV benchmark dataset (Jones *et al.*, 2015). To test EigenTHREADER’s tolerance to sparse or low quality data we generated 8 additional contact subsets taking only a proportion of the contacts for each dataset. For one experiment, we took the top scoring L (sequence length), L/2, L/5 and L/10 long range contacts (sequence separation >21 residues). For the other experiment the lists of contacts for each lists were randomized rather than ranked by prediction score, we then took an L, L/2, L/5 and L/10 set of long range contacts from these randomized lists.

### 2.5 Benchmark comparison software

EigenTHREADER performance was benchmarked against the state-of-the-art fold recognition methods HHSuite 3.0.0 (https://github.com/soedinglab/hh-suite) and pGenTHREADER 8.9 (http://bioinfadmin.cs.ucl.ac.uk/downloads/pGenTHREADER/).

#### 2.5.1 Fold and sequence libraries

To perform a valid comparison between EigenTHREADER, HHSearch and pGenTHREADER, identical fold libraries were constructed. We downloaded the 13,730 HHSearch a3m files for SCOP 1.75 (http://www.user.gwdg.de/∼compbiol/data/hhsuite/databases/hhsearch_dbs/). These were used to prepare the relevant HH-Suite Hidden Markov Models and library files as per the HH-Suite documentation. For each HH-Suite SCOP a3m file we constructed the equivalent fold library files for EigenTHREADER and pGenTHREADER. We note that we could not generate EigenTHREADER fold library files for a trivial number of the 13 730 domains resulting in a slightly smaller database of 13 613 domains. To maintain parity between each of our fold libraries we deleted these ‘missing’ entries in the EigenTHREADER library from the HHSearch library such that all three libraries cover the same set of 13,613 domains.

Uniref90 (Suzek *et al.*, 2015) for the pGenThreader PSIBLAST was downloaded from UniProt FTP server and for the HHBlits profile generation we downloaded the uniprot20_2013_03 sequence database.

Additionally, we wanted to investigate EigenTHREADER runtimes. A fold library based on whole PDB chains (Berman *et al.*, 2000), 12 833 chains, rather than domains was prepared to represent a potential worst-case runtime use of EigenTHREADER.

## 3 Results

EigenTHREADER has several tuneable parameters, two of which are performance critical: the number of eigenvectors to match and the contact distance. To find the optimal values for each of these parameters we generated EigenTHREADER predictions across the whole benchmark dataset holding one of the two parameters constant and incrementing the value of the test parameter in integer steps. We ran a non-exhaustive search for both parameters with the number of eigenvectors tested from 1 to 20 (contact distance held at 10 Å) and contact distances from 1 to 20 Å tested (eigenvectors held at 20). As both parameters are unlikely to have any non-linear interaction a grid search of these parameters was not conducted.

### 3.1 EigenTHREADER runtimes

**Fig. 1:**
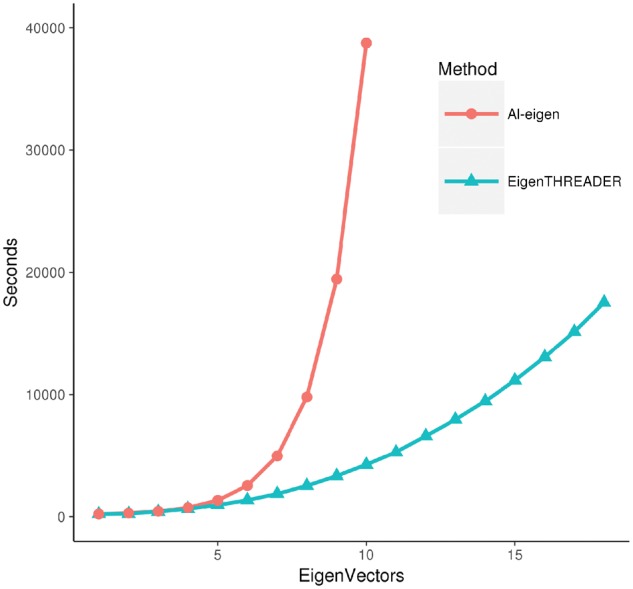
EigenTHREADER and Al-eigen runtimes. Average runtime in seconds as a function of the number of eigenvectors used. The contact fold library used contained 12,833 full length PDB chains. Al-eigen runtimes are estimated ater the paper of Di Lena *et al*

Figure 1 shows the increase in runtime as the number of eigenvectors is increased. Increasing the number of eigenvectors brings with it increased fold recognition performance, but trading off a quadratic increase in runtime. It is worth noting that as the size of the fold library is increased, runtimes scale linearly as the time to match each fold library entry is approximately constant for a given number of eigenvectors (data not shown). Alongside the EigenTHREADER runtimes we show the estimated runtimes for Al-eigen given the exponential increase in runtime reported in the work of Di Lena *et al.* It is clear that EigenTHREADER represents a substantial increase in performance.

### 3.2 Impact of the number of eigenvectors on fold recognition performance

**Fig. 2:**
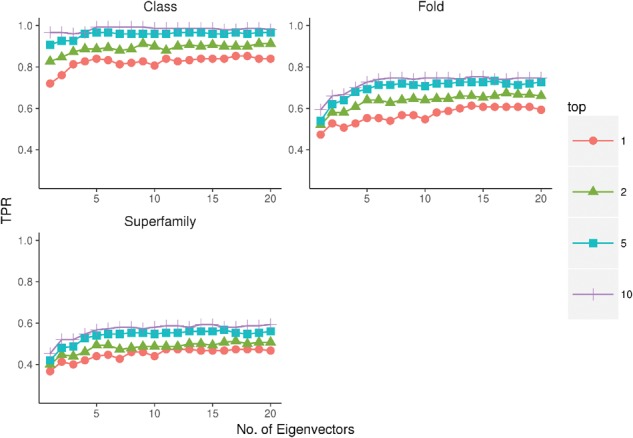
Performance as number of eigenvectors increase. Average True Positive Rate of predictions for the 150 benchmark proteins for EigenTHREADER as the number of eigenvectors is adjusted from 1 to 20. Plots show the performance for SCOP Class, Fold and Superfamily predictions considering only the top 1, 2, 5 or 10 scoring predictions

In Figure 2 we show the true positive rate as a function of the number of eigenvectors. Performance is broken down on a t1, t2, t5 and t10 basis, where a true positive has been counted if the correct Class, Fold or Superfamily is found anywhere in the top 1, 2, 5 or 10 results. For all three prediction levels, as we relax the true positive stringency (t1 to t10) the recognition performance increases, as expected. When predicting SCOP class there is no substantial increase in performance as the number of eigenvectors increases, indicating that all the information available for such a prediction is contained in the first eigenvector. At the fold and superfamily levels, as the number of eigenvectors increases the performance also increases. This is expected as each eigenvector should add increasing information to the prediction and there ought to be additional information beyond the first eigenvector. Performance is seen to level out at around 10 eigenvectors but we assume performance increases should slowly continue past 20 eigenvectors. We stopped at 20, as run times begin to become prohibitive for trivial increases in performance.

### 3.3 Impact of contact distance on performance

**Fig. 3:**
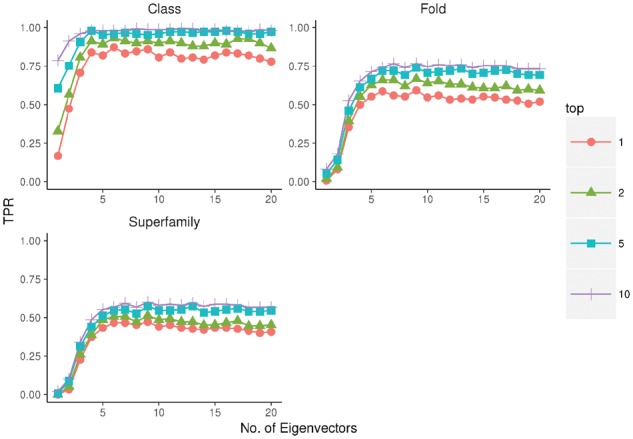
Performance as the distance threshold is increased. Average True positive rate of predictions for the 150 benchmark proteins as the EigenTHREADER distance threshold is adjusted from 1 to 20. Plots show the performance for SCOP Class, Fold and Superfamily predictions considering only the top 1, 2, 5 or 10 scoring predictions

Figure 3 shows change in performance as we adjust the contact distance parameter. In all cases, there is little predictive power when only contacts below 5 Å are included. Performance rapidly increases as the contact distance increases reaching peak performance between 7 and 10 Å. Performance tails off once the contact distance exceeds 11 or 12 Å. This is consistent with the distance thresholds found to be optimal for contact-assisted *de novo* folding (Nugent and Jones, 2012; Kosciolek and Jones, 2014).

### 3.4 Performance with sparse data

**Fig 4:**
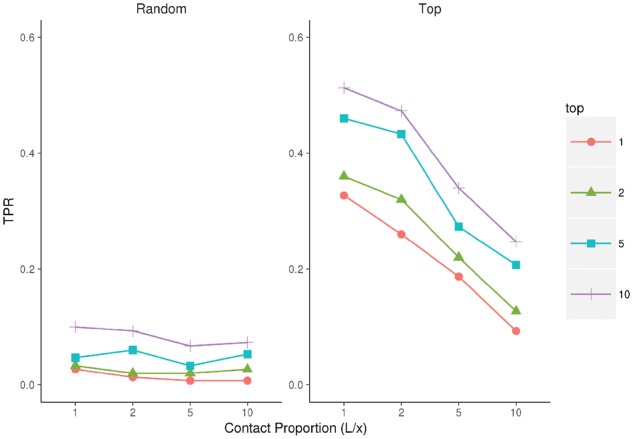
EigenTHREADER fold prediction performance. Fold prediction using the Top or Random L, L2, L5 or L10 MetaPSICOV contacts. Comparison shows TPR performance when considering either the top 1, 2, 5 or 10 EigenTHREADER predictions

Figure 4 shows the fold prediction results when running EigenTHREADER with very sparse, long range contact data with either the most confident predictions (Top) or a random set of predictions (Random), see Section 2.4. As expected, as the number of predictions becomes exceedingly sparse, moving from L to L/10, the TPR rate declines rapidly. This correlates to moving from using only 1–5% of the most confident MetaPSICOV predictions to using less than 0.15% of the top contacts. When considering the Top L predictions, the TPR is about 0.2 lower than the peak performances seen in Figures 2 and 3 using only one 20th of the data. This indicates that EigenTHREADER predictions are still robust even with little contact data available. As a control, when randomized contacts are used, it’s clear that EigenTHREADER performs poorly, as expected, indicating the importance of obtaining correct, high quality contact data for correct fold recognition.

### 3.5 Comparison of EigenTHREADER, pGenTHREADER and HHSearch

#### 3.5.1 Analogous fold recognition

**Fig. 5:**
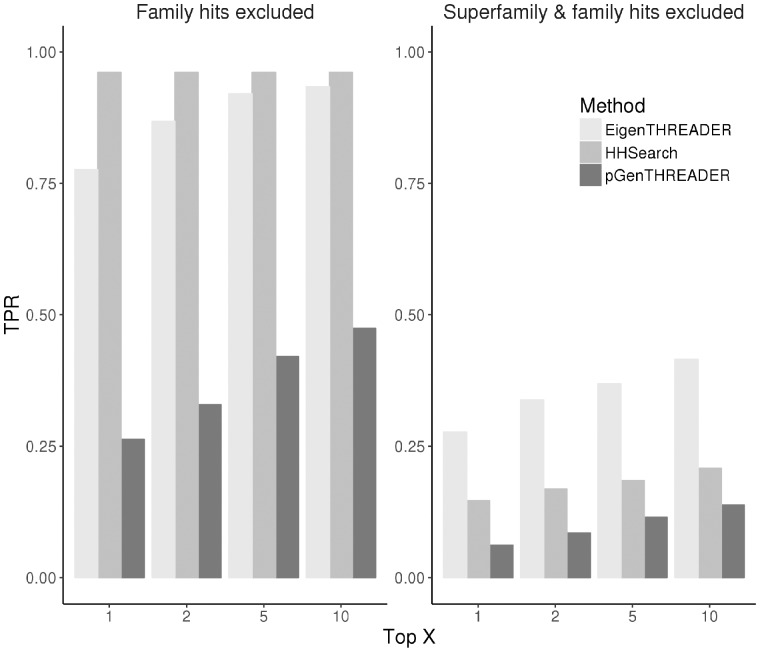
True Positive Rate comparison for analogous fold recognition. Average True Positive Rate performance for EigenTHREADER, pGenTHREADER and HHSearch across the benchmark target proteins. For these fold recognition searches, the left-hand bar chart considers only matches at fold and superfamily levels (calculated over 130 benchmark proteins). The right-hand bar chart considers matches only at the fold level (calculated over 76 benchmark proteins)

EigenTHREADER was developed to enable fold recognition in instances where homology based fold recognition is not possible. We have compared the performance of EigenTHREADER in this specific task with two other widely used fold recognition methods; pGenTHREADER and HHSearch. pGenTHREADER is a profile-profile search method which compares a sequence profile generated with PSIBLAST against a library of structure profiles. In the HHSearch case we first used HHBlits to generate sequence profile HMMs and then used these to search the fold library using HHSearch. We are also interested using such predictions to build high quality models, any hits that have less than 40% overlap with the query sequence were also excluded. Figure 5 shows the average true positive rate for the top 1, top 2, top 5 and top 10 predictions for each prediction method. For the following analysis, we have excluded any hits which shared the same SCOP family (left-hand bar chart) or where SCOP family and superfamily are excluded (right-hand bar chart). When family and super family members are excluded it reduces the number of benchmark proteins where a True Positive is attainable. Where family hits are excluded the TPR is calculated over 130 benchmark proteins, when both superfamily and family hits are excluded the TPR is calculated over only 76 proteins. The left-hand bar chart simulates the case where there is minimal homology information present in the fold library for each benchmark protein. The right-hand bar chart simulates the case where there are no homologous relatives for each benchmark protein in the fold library. These highly stringent criteria eliminate most hits from the results of all three methods.

We see that HHSearch outperforms both pGenTHREADER and EigenTHREADER when homology is present in the fold library (left-hand bar chart). This is to be expected as we know that HHSearch is among the most sensitive sequence homology searching methods available today. However, when we exclude SCOP Superfamily and Family matches from the results the performance of all three methods more than halves. In this case EigenTHREADER shows better performance than the other two methods, nearly 4 times the performance of pGenTHREADER and about twice that of HHSearch. This indicates the EigenTHREADER can have a role to play in the instances of fold recognition where no homologues can be found.

#### 3.5.2 Model quality comparison

All three methods compared are able to produce low resolution backbone structural models based on the fold alignments obtained during the searches (see Section 3.5.1). Under our stringent filtering criteria we note that only 103 of the benchmark proteins find suitable structural templates via HHSearch. The structure comparison scores are calculated only over this subset.

Table 1 summarizes the TM-score (Zhang and Skolnick, 2005) and GDT-TS (Zemla *et al.*, 1999) scores for the best models created by the three methods. Models generated by EigenTHREADER for analogous hits outperform those produced by both pGenTHREADER and HHSearch, for the 123 benchmark proteins which HHSearch finds hits. As we move from T1 to T5 the average median scores typically fall as the model variability rises as more models with lower scores are included in the statistic. The averaged TM and GDT max scores are seen to increase for all methods, indicating that the best fitting model is not always the highest scoring hit.

**Table 1:** Median and best max TM-score and GDT-TS scores

	EigenTHREADER		pGenTHREADER			HHSearch	
	TM-score (**median**/max)	GDT-TS	TM-score		GDT-TS	TM-score	GDT-TS
T1	**0.35/**0.35	**29.47/**29.47	**0.19/**0.19	**16.25**/16.25		**0.19/**0.19	**18.5**/18.5
T5	**0.32**/0.39	**28.04**/33.1	**0.18**/0.23	**16.03**/19.44		**0.18**/0.19	**18.63/**22.37

The table gives the median TM-Score and GDT-TS score for the Top (highlighted) and Top 5 hits across benchmark set alongside the best score achieved by any target. Values are averaged over 103 benchmark proteins.

**Fig. 6:**
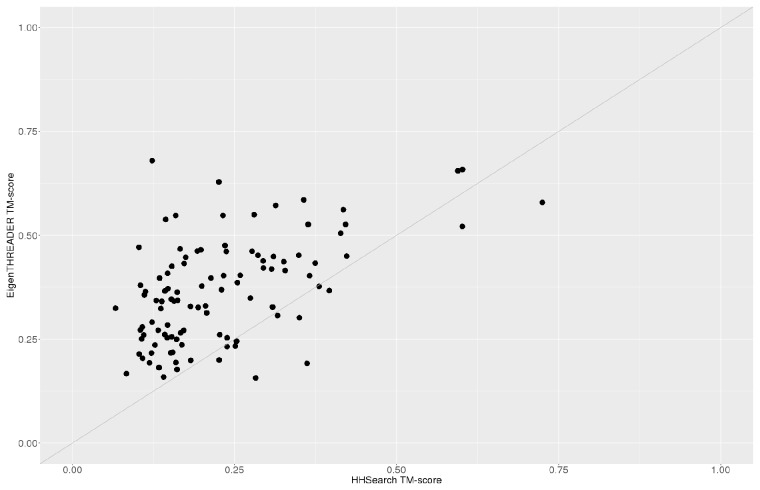
Comparison of EigenTHREADER and HHSearch T1 TM scores. Each point represents a single benchmark protein. The TMscore (x and y axes) for the highest scoring model for both methods are plotted

In Figure 6 we plot the actual TM scores of the T1 hits from both EigenTHREADER and HHSearch for the benchmark proteins. Nearly all the EigenTHREADER T1 models have greater TM scores than the HHSearch T1 models. This indicates that EigenTHREADER’s best hit template is either closer to the target structure for that benchmark protein, or that the alignment to the template may be more accurate.

## 4 Conclusion

In the presence of detectable homologous structures, protein fold recognition may be regarded as being a mostly solved problem. Previous results amply demonstrate that methods such as pGenTHREADER and HHSearch achieve very high accuracy for this aspect of the fold recognition problem. Recognition of analogous folds, where no homologues exists in the fold library, is anything but a solved problem. Performance of predictive methods in this task, is typically poor. In this paper we have presented and benchmarked an alternative approach to fold recognition, EigenTHREADER, which relies only on residue contacts predicted from sequence alignments. Our benchmark demonstrates that EigenTHREADER outperforms both pGenTHREADER and HHSearch in the challenging task of analogous fold recognition, although it is not as sensitive in the task of homologous fold search. This work further demonstrates the power of recently developed co-evolutionary contact prediction methods in varied structural bioinformatics applications. Given the ability to predict an accurate contact map, and assuming the native fold is present in the fold library, EigenTHREADER offers an alternative path to identify useful templates for homology modelling. This should make template-based modelling a viable option for many more structurally uncharacterized sequence families in the near future.

## References

[btx217-B1] Berman H.M. et al (2000) The Protein Data Bank. Nucleic Acids Res., 28, 235–242. 10592235 10.1093/nar/28.1.235 PMC102472

[btx217-B2] Creighton T.E. (1992) Proteins: Structures and Molecular Properties. New York, NY, W. H. Freeman.

[btx217-B3] Di Lena P. et al (2010) Fast overlapping of protein contact maps by alignment of eigenvectors. Bioinformatics, 26, 2250–2258. 20610612 10.1093/bioinformatics/btq402

[btx217-B4] Gniewek P. et al (2014) BioShell-Threading: versatile Monte Carlo package for protein 3D threading. BMC Bioinformatics, 15, 22. 24444459 10.1186/1471-2105-15-22 PMC3937128

[btx217-B5] Goldman D. (1999) Algorithmic aspects of protein structure similarity**.** In: FOCS 1999 Proceedings of the 40th Annual Sumposium on Foundation of Computer Science, 512–521.

[btx217-B6] Jones D.T. et al (2012) PSICOV: precise structural contact prediction using sparse inverse covariance estimation on large multiple sequence alignments. Bioinformatics, 28, 184–190. 22101153 10.1093/bioinformatics/btr638

[btx217-B7] Jones D.T. et al (2015) MetaPSICOV: combining coevolution methods for accurate prediction of contacts and long range hydrogen bonding in proteins. Bioinformatics, 31, 999–1006. 25431331 10.1093/bioinformatics/btu791 PMC4382908

[btx217-B8] Jones D.T. et al (1992) A new approach to protein fold recognition. Nature, 358, 86–89. 1614539 10.1038/358086a0

[btx217-B9] Kaján L. et al (2014) FreeContact: fast and free software for protein contact prediction from residue co-evolution. BMC Bioinformatics, 15, 85. 24669753 10.1186/1471-2105-15-85 PMC3987048

[btx217-B10] Kinch L.N. et al (2016) Assessment of CASP11 contact-assisted predictions. Proteins, 84, 164–180. 26889875 10.1002/prot.25020 PMC5485253

[btx217-B11] Kosciolek T., Jones D.T. (2014) De novo structure prediction of globular proteins aided by sequence variation-derived contacts. PLoS One, 9, e92197. 24637808 10.1371/journal.pone.0092197 PMC3956894

[btx217-B12] Lobley A. et al (2009) pGenTHREADER and pDomTHREADER: new methods for improved protein fold recognition and superfamily discrimination. Bioinformatics, 25, 1761–1767. 19429599 10.1093/bioinformatics/btp302

[btx217-B13] Ma J. et al (2014) MRFalign: protein homology detection through alignment of Markov random fields. PLoS Comput. Biol., 10, e1003500. 24675572 10.1371/journal.pcbi.1003500 PMC3967925

[btx217-B14] Ma J. et al (2013) Protein threading using context-specific alignment potential. Bioinformatics, 29, i257–i265. 23812991 10.1093/bioinformatics/btt210 PMC3694651

[btx217-B15] Marks D.S. et al (2011) Protein 3D structure computed from evolutionary sequence variation. PLoS One, 6, e28766. 22163331 10.1371/journal.pone.0028766 PMC3233603

[btx217-B16] Mitchell S.D., Gronenborn A.M. (2015) After fifty years, why are protein X-ray crystallograpers still in business? Br. J. Philos. Sci., 66, 1–21.

[btx217-B17] Moult J. et al (2014) Critical assessment of methods of protein structure prediction (CASP)–round x. Proteins, 82, 1–6. 10.1002/prot.24452 PMC4394854 24344053

[btx217-B18] Needleman S.B., Wunsch C.D. (1970) A general method applicable to the search for similarities in the amino acid sequence of two proteins. J. Mol. Biol., 48, 443–453. 5420325 10.1016/0022-2836(70)90057-4

[btx217-B19] Nugent T., Jones D.T. (2012) Accurate de novo structure prediction of large transmembrane protein domains using fragment-assembly and correlated mutation analysis. Proc. Natl. Acad. Sci. U. S. A., 109, E1540–E1547. 22645369 10.1073/pnas.1120036109 PMC3386101

[btx217-B20] Olmea O. et al (1999) Effective use of sequence correlation and conservation in fold recognition. J. Mol. Biol., 293, 1221–1239. 10547297 10.1006/jmbi.1999.3208

[btx217-B21] Peng J., Xu J. (2011) A multiple-template approach to protein threading. Proteins, 79, 1930–1939. 21465564 10.1002/prot.23016 PMC3092796

[btx217-B22] Rost B. et al (1997) Protein fold recognition by prediction-based threading. J. Mol. Biol., 270, 6471–6480. 10.1006/jmbi.1997.1101 9237912

[btx217-B23] Seemayer S. et al (2014) CCMpred–fast and precise prediction of protein residue-residue contacts from correlated mutations. Bioinformatics, 30, 3128–3130. 25064567 10.1093/bioinformatics/btu500 PMC4201158

[btx217-B24] Söding J. (2005) Protein homology detection by HMM-HMM comparison. Bioinformatics, 21, 6951–6960. . 10.1093/bioinformatics/bti125 15531603

[btx217-B25] Söding J., Remmert M. (2011) Protein sequence comparison and fold recognition: progress and good-practice benchmarking. Curr. Opin. Struct. Biol., 21, 6404–6411. 10.1016/j.sbi.2011.03.005 21458982

[btx217-B26] Strang G. (2016) Introduction to Linear Algebra, 5th ed., Wellesley, MA, Cambrdige Press.

[btx217-B27] Suzek B.E. et al (2015) UniRef clusters: a comprehensive and scalable alternative for improving sequence similarity searches. Bioinformatics, 31, 926–932. 25398609 10.1093/bioinformatics/btu739 PMC4375400

[btx217-B28] Wu S., Zhang Y. (2008) MUSTER: improving protein sequence profile-profile alignments by using multiple sources of structure information. Proteins, 72, 547–556. 18247410 10.1002/prot.21945 PMC2666101

[btx217-B29] Zemla A. et al (1999) Processing and analysis of CASP3 protein structure predictions. Proteins, 37, 22–29. 10.1002/(sici)1097-0134(1999)37:3+<22::aid-prot5>3.3.co;2-n 10526349

[btx217-B30] Zhang Y., Skolnick J. (2005) TM-align: a protein structure alignment algorithm based on the TM-score. Nucleic Acids Res., 33, 2302–2309. 15849316 10.1093/nar/gki524 PMC1084323

[btx217-B31] Zhou H., Zhou Y. (2005) Fold recognition by combining sequence profiles derived from evolution and from depth-dependent structural alignment of fragments. Proteins, 58, 6321–6328. 10.1002/prot.20308 PMC1408319 15523666

